# *ist-1/IRS1* affects L1 starvation resistance in *daf-16/FoxO-* dependent and independent fashion

**DOI:** 10.17912/micropub.biology.001648

**Published:** 2025-06-13

**Authors:** Jingxian Chen, Ainsley R. Scheiner, Ivan B. Falsztyn, L. Ryan Baugh

**Affiliations:** 1 Department of Biology, Duke University; 2 Department of Biology, University Program in Genetics and Genomics, Duke University

## Abstract

The mammalian IRS1 gene is an important adaptor for the insulin and insulin-like growth factor receptors, but its sole homolog in the nematode *C. elegans* , *ist-1* , has received relatively little attention. We show that *ist-1 /IRS1* has modest effects on L1 starvation resistance, with two null mutants increasing larval growth and reproduction after recovery from extended L1 arrest. *ist-1 /IRS1* mutants increase nuclear localization of DAF-16 /FoxO, a critical effector of insulin/IGF signaling, in starved L1 larvae, consistent with IST-1 /IRS1 transducing DAF-2 /IGFR signaling. However, epistasis analysis suggests that *ist-1 /IRS1* also functions independently of *daf-16 /FoxO* , suggesting additional, novel function.

**Figure 1. ist-1/IRS1 affects L1 starvation resistance and DAF-16::GFP nuclear localization, but it also functions independently of daf-16/FoxO:**
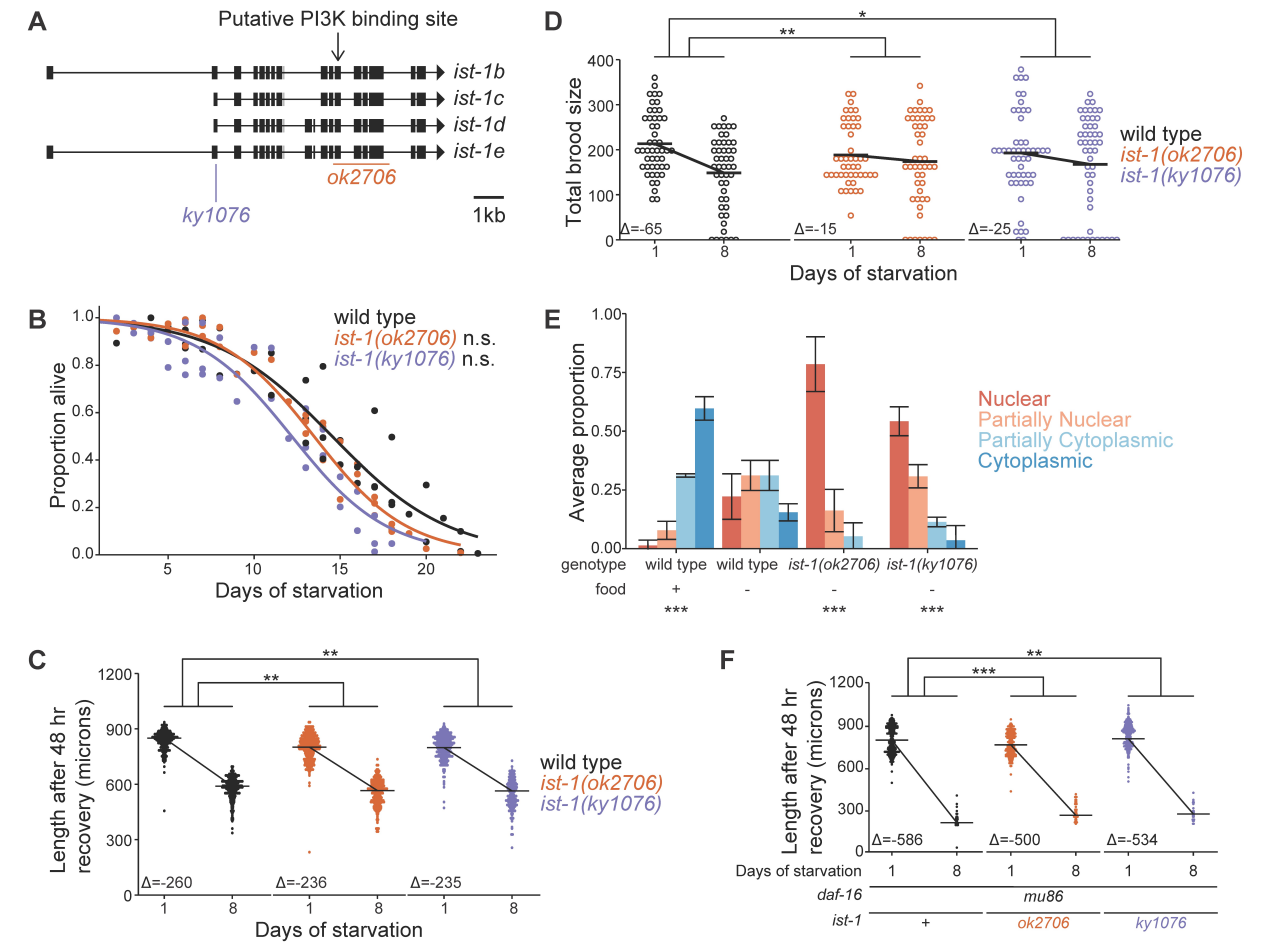
A) Schematic of the *ist-1* gene (adapted from (Cheng et al., 2022)). B) L1 starvation survival. Proportion alive was scored daily. For each genotype and each replicate, quasi-binomial logistic regression was performed with the response variable being proportion alive and the explanatory variable being duration of L1 starvation. Half-lives were estimated from the regression. Unpaired, variance-pooled t-tests were performed using half-lives comparing each mutant to wild type. C, F) Length after 48 hr recovery from 1 or 8 d L1 arrest in wild-type (C) or *daf-16 /FoxO* null (F) background. D) Total brood size after recovery from 1 or 8 d L1 arrest. E) DAF-16 ::GFP subcellular localization in L1 larvae. Larvae hatched in the presence (~6 hr) or absence (~12 hr) of food, and four categories of localization were scored in intestinal cells. Error bars represent standard deviations. Cochran–Mantel–Haenszel test was performed comparing starved wild type to each of the other conditions. C, D, F) A linear mixed-effects (lme) model was used (body length (C and F) or total brood size (D) ~ genotype * days of starvation, random effect = replicates) to compare each mutant to wild type. Interaction p-values are presented, effectively testing for differences in slope of lines connecting body length or total brood size estimates from lme. Horizontal bars represent body length or total brood size estimated by lme. B-F) At least three biological replicates were performed. *P<0.05, **P<0.01, ***P<0.001, n.s. not significant.

## Description

Insulin signaling is highly conserved among metazoans and links nutrient availability to development, metabolism, stress resistance, and cancer (Bose et al., 2020; Hopkins et al., 2020; Suzawa & Bland, 2023). In mammals, activation of the insulin receptor (IR) and insulin-like growth factor receptor (IGFR) leads to phosphorylation of insulin receptor substrate (IRS) proteins, which act as intermediates to relay signals -- primarily through the phosphoinositide 3-kinase (PI3K) signaling cascade (Shaw, 2011). Much has been learned about insulin/IGF signaling (IIS) in *Caenorhabditis elegans* (Murphy & Hu, 2013), but the role of IRS proteins is unclear. IST-1 /IRS1 contains predicted phospholipid-binding pleckstrin homology (PH), phosphotyrosine-binding (PTB), and PI3K-binding domains (Wolkow et al., 2002). However, unlike mammalian IRS proteins, which possess multiple YxxM motifs critical for PI3K binding, IST-1 has only a single YxxM motif. This difference suggests that IST-1 may play a marginal role in mediating PI3K activation by DAF-2 /IGFR. Indeed, *ist-1* mutants do not exhibit the temperature-sensitive dauer-formation constitutive phenotype seen in *daf-2 /IGFR* and *age-1 /PI3K* mutants (Murphy & Hu, 2013), though *ist-1* RNAi enhances dauer formation in an *age-1 /PI3K* mutant background (Wolkow et al., 2002). In addition, *ist-1 /IRS1* is required for aversive olfactory learning, like *daf-2 /IGFR* and *age-1 /PI3K* , and it appears to function upstream of *age-1* (Cheng et al., 2022). *daf-16 /FoxO* , a critical effector of IIS in regulation of dauer formation and lifespan, is epistatic to *daf-2 /IGFR* and *age-1 /PI3K* (Murphy & Hu, 2013), but, surprisingly, *ist-1 /IRS1* function in aversive olfactory learning is partially independent of *daf-16 /FoxO* (Cheng et al., 2022) *.* IIS also regulates starvation resistance during L1 arrest (Baugh, 2013). Here, we report that *ist-1 /IRS1* affects starvation resistance by modulating IIS but that it also functions independently of *daf-16 /FoxO* .

We analyzed two putative null *ist-1 /IRS1* mutants: *ok2706* , an 1873 bp deletion that disrupts the putative PI3K binding motif, and *ky1076* , a 5 bp insertion at the beginning of the first exon shared by all *ist-1* isoforms ( Fig. 1A ) (Cheng et al., 2022). Neither mutant affected L1 starvation survival ( Fig. 1B ). However, starvation resistance also involves the ability to recover and reproduce, and there are mutants that do not affect survival but do affect growth and reproduction upon feeding (Baugh & Hu, 2020). The relative effect of extended starvation (8 d) on larval growth and reproduction was mitigated in both *ist-1* mutants ( Fig. 1C, D). That is, both mutants grew slower and produced smaller broods after 8 d L1 arrest compared to 1 d (relatively brief arrest to synchronize populations), but the effect of starvation on these traits was significantly reduced in the mutants (evident in the slope of the lines connecting 1 and 8 d starvation). *daf-2 /IGFR* mutants display a similar but greater suppression of the effects of extended starvation (Falsztyn et al., 2025), and disruption of *daf-2* also increases starvation survival (Baugh & Sternberg, 2006; Munoz & Riddle, 2003). Given the function of mammalian IRS proteins, these results suggest that IST-1 /IRS1 transduces IIS to limit starvation resistance, though its effect is relatively modest compared to *daf-2 /IGFR* and other core components of the IIS pathway.

DAF-2 /IGFR activates the AGE-1 /PI3K signaling cascade to antagonize the transcription factor DAF-16 /FoxO, thereby promoting growth and development (Murphy & Hu, 2013). However, during starvation IIS is reduced, and DAF-16 /FoxO enters the nucleus and activates transcription of genes that promote developmental arrest and survival (Baugh & Hu, 2020). We hypothesized that if *ist-1 /IRS1* functions as a mediator of DAF-2 /IGFR signaling, then it should also antagonize DAF-16 /FoxO activity. We assayed DAF-16 ::GFP nucleocytoplasmic localization as a proxy for DAF-16 activity. Starved L1 larvae displayed more DAF-16 ::GFP nuclear localization than fed L1 larvae ( Fig. 1E ), as expected. Furthermore, both *ist-1 /IRS1* mutants exhibited a higher proportion of nuclear DAF-16 ::GFP than wild type during starvation ( Fig. 1E ), consistent with IST-1 /IRS1 functioning as a mediator of DAF-2 /IGFR signaling in starved L1 larvae.

We used epistasis analysis to determine if loss of *ist-1 /IRS1* requires *daf-16 /FoxO* to increase L1 starvation resistance. Surprisingly, both *ist-1 /IRS1* mutants mitigated the effect of extended starvation on larval growth upon recovery in a *daf-16 /FoxO* null mutant background ( Fig. 1F ). Although *ist-1 /IRS1* appears to transduce IIS and affect DAF-16 /FoxO activity ( Fig. 1E ), these results suggest that *ist-1 /IRS1* also functions independently of *daf-16 /FoxO* to affect L1 starvation resistance.

In summary, we show that *ist-1 /IRS1* modestly inhibits L1 starvation resistance. We also show that *ist-1 /IRS1* antagonizes nuclear localization of DAF-16 /FoxO, consistent with *ist-1 /IRS1* transducing IIS, as it is thought to do in dauer formation and aversive olfactory learning. However, the effects of *ist-1 /IRS1* on starvation resistance (and dauer formation) are relatively mild compared to what is seen for mammalian IRS genes. Furthermore, our results suggest that *ist-1 /IRS1* also functions independently of *daf-16 /FoxO* , as in aversive olfactory learning, suggesting that *ist-1 /IRS1* regulates starvation resistance through one or more additional mechanisms. It is possible that IST-1 /IRS1 affects an effector of PI3K signaling other than DAF-16 /FoxO ( *e.g.* , SKN-1 /Nrf or mTOR). Alternatively, IST-1 /IRS1 could function beyond transducing activating signals from DAF- 2/IGFR to AGE-1 /PI3K. Notably, we focused on L1 arrest and recovery, and it is unclear what additional, presumably subtle, phenotypes *ist-1 /IRS1* may have or whether *ist-1 /IRS1* affects DAF-16 /FoxO nuclear localization in fed larvae or other developmental stages.

## Methods

*C. elegans* maintenance

All strains were maintained with *E. coli* OP50 on nematode growth medium plates (NGM) and were well-fed for at least three generations before being used in experiments. Worms were cultured and starved at 20°C. N2 is from the Sternberg collection, originally received from the CGC in 1987.

Starvation survival

Seven L4-stage worms were transferred to a 10 cm NGM plate with an OP50 lawn, with four plates per strain. Worms were cultured for 96 hr before they were hypochlorite treated ("bleached") to prepare embryos (Hibshman et al., 2021). Embryos were resuspended, washed, counted, and cultured in S-basal with 0.15% ethanol. Cultures had 1 embryo/µL in 5 mL and were placed in 16 mm glass tubes on a tissue-culture roller drum so embryos hatch and enter L1 arrest (Hibshman et al., 2021). The day after bleaching, and again every day after that, a 100 µL aliquot of each starvation culture was plated on a 6 cm NGM plate beside an OP50 lawn. The number of larvae plated was counted (total plated). Two days later, the number of live worms on the lawn was counted (total alive). Proportion alive was determined as total alive divided by total plated.

Larval size

Strains were treated as for Starvation survival except that cultures did not contain ethanol. 500 µL of starvation culture was plated on 10 cm NGM plates seeded with OP50 1 or 8 d after setting up starvation cultures, then recovered for 48 hr. Worms were washed off plates with virgin S-basal and transferred to unseeded 10 cm NGM plates for imaging using ZEISS SteREO Discovery.V20. The image-analysis program WormSizer was used to determine worm length (Moore et al., 2013).

Brood size

Strains were treated as for Larval size. After being recovered for 48 hr following L1 arrest, 18 larvae were randomly selected and individually transferred to 6 cm NGM plates with OP50 (one worm per plate). Worms were transferred to a new plate each day until egg laying ceased. Two days after transfer, the number of progeny laid was counted. Total brood size equals the sum of progeny laid across all plates for a given worm.

DAF-16 ::GFP localization

Starved strains were treated as described in Starvation survival except that cultures did not contain ethanol. Fed samples were prepared by plating embryos on NGM plates with OP50 after bleaching. Fed L1s were examined 18 hours after plating embryos (~6 hr after hatching) (Chen et al., 2025). Starved L1s were examined 24 hours after setting up starvation cultures (~12 hr after hatching). Worms were collected in 1.5 mL centrifuge tubes, washed with virgin S-basal, and centrifuged at 3,000 rpm for 30 sec. 1.5 µL of worm pellet was pipetted onto the center of a slide with a 4% Noble agar pad, and a glass cover slip was immediately placed on top. A timer was set for 3 min, and the slide was systematically scanned with each individual worm scored for nucleocytoplasmic localization specifically within intestinal cells at 1000x on a Zeiss Axio Imager A1. Nucleocytoplasmic localization of DAF-16 ::GFP was assigned to one of four categories: nuclear, partially nuclear, partially cytoplasmic, and cytoplasmic. Scoring for each slide stopped after 3 min to avoid confounding effects of worms being mounted on slides.

## Reagents

**Table:** 

STRAIN	GENOTYPE	RECEIVED FROM	AVAILABLE FROM
N2	Wild type	Sternberg lab, Caltech	CGC
CF1038	*daf-16 ( mu86 ) I *	CGC	CGC
CX1076	*ist-1 (ky1076) X *	Bargmann lab, Rockefeller	Bargmann lab, Rockefeller
CX17790	*ist-1 ( ok2706 ) X *	Bargmann lab, Rockefeller	Bargmann lab, Rockefeller
OH16024	*daf-16 ( ot971 [ daf-16 ::GFP]) I *	Hobert lab, Columbia	CGC
LRB590	*daf-16 ( ot971 [ daf-16 ::GFP]) I; * *ist-1 (ky1076) X *	Generated	Baugh lab, Duke
LRB591	*daf-16 ( ot971 [ daf-16 ::GFP]) I; * *ist-1 ( ok2706 ) X *	Generated	Baugh lab, Duke
LRB660	*daf-16 ( mu86 ) I; ist-1 (ky1076) X *	Generated	Baugh lab, Duke
LRB661	*daf-16 ( mu86 ) I; ist-1 ( ok2706 ) X *	Generated	Baugh lab, Duke
